# A Unique Case of a Massive Fungating Fibroadenoma of the Breast: Clinical and Pathological Insights

**DOI:** 10.7759/cureus.71375

**Published:** 2024-10-13

**Authors:** Dalal A Yusuf, Alya Wael, Sara George, Raja Eid

**Affiliations:** 1 General Surgery, Salmaniya Medical Complex, Manama, BHR; 2 Orthopedics, Salmaniya Medical Complex, Manama, BHR; 3 Pathology and Laboratory Medicine, Salmaniya Medical Complex, Manama, BHR

**Keywords:** benign breast lesion, fungating mass, giant juvenile fibroadenoma, leiomyoma, phyllodes tumor

## Abstract

Fibroadenoma is a type of benign tumor that occurs in breast tissues, commonly in younger age groups. However, the presentation of the patient described below shows how the disease presents in various manners. A 52-year-old woman presented to the outpatient clinic with a unilateral, giant, left-sided fungating breast mass who was later admitted under the General Surgical team, at Salmaniya Medical Complex (SMC) for further corresponding diagnostic workup. A staging CT scan of the abdomen/pelvis and chest/thorax showed a left, large, heterogeneously enhancing, fungating breast lesion, involving most breast quadrants measuring around 17 × 12 cm with an unexpected hamartoma in the right breast. An incidental finding of leiomyoma was found on the left lateral border of the uterine cervix. Diagnosis of giant fibroadenoma was established only after the surgical management was completed. Management of this case was approached by multidisciplinary surgical teams. We are reporting this case to highlight the significance of the breast screening protocols, hence avoiding late and complicated presentations of the disease. This case report is one of few, which will help future researchers formulate precise guidelines dedicated to huge fibroadenomas.

## Introduction

Fibroadenomas are benign, encapsulated, and circumscribed lesions typically found in adolescent to young women aged 14 to 35 years, rarely seen in post-menopausal women [1]. A giant fibroadenoma is defined as one larger than 5 cm or weighing over 500 g [2,3]. Only 0.5% to 2% of benign fibroadenomas are classified as giant, primarily occurring in pregnant or lactating women and some adolescents [1-3]. It is reported that 25% of female patients experience non-giant benign fibroadenomas, which are usually asymptomatic [4].

The pathophysiology of giant fibroadenomas involves genetic factors and hormonal changes, particularly in those on oral contraceptives or who are pregnant [3]. A significant diagnostic challenge lies in differentiating between phyllodes tumors and giant fibroadenomas due to their similar molecular and morphological characteristics [5]. Giant juvenile fibroadenomas are often misdiagnosed as phyllodes tumors, physiological hypertrophy, or breast abscesses due to their rarity [6]. Because phyllodes tumors present homogeneously and share histological similarities with fibroadenomas, surgeons may perform a core biopsy to rule out malignancy [7].

The presence of a rapidly growing mass that ulcerates the skin, along with a tendency to recur, raises suspicion of malignancy [8]. In this case report, we present a 52-year-old woman from Southeast Asia diagnosed with a left breast giant fibroadenoma. We will discuss her unusual presentation and the management of this late-presenting condition.

## Case presentation

**Figure 1:**
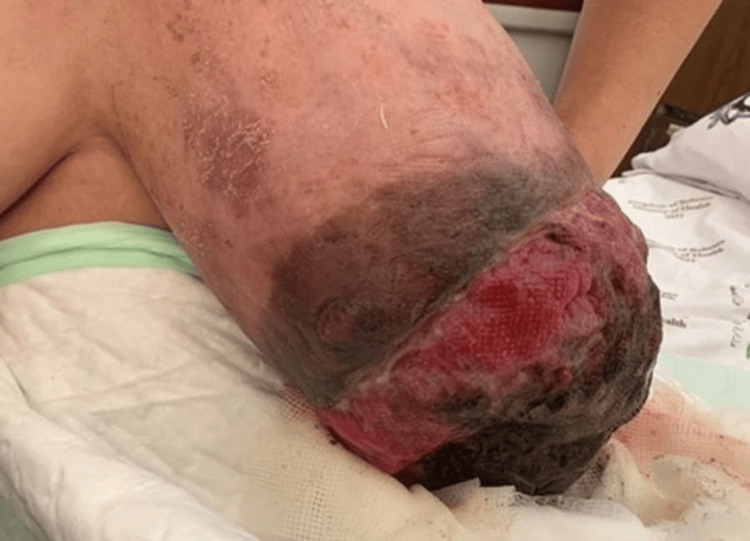
Left fungating breast mass

A 52-year-old married woman with no significant medical history presented to the breast clinic at Salmaniya Medical Complex. Her chief complaint was a rapidly growing mass in her left breast, which had developed over the past nine months and began to fungate two weeks prior to her visit (Figure 1).

The patient reported experiencing intermittent needle-prick sensations in her left breast. She had regular menstrual cycles, with menarche at age 11, and experienced menorrhagia without dysmenorrhea starting at age 49. There was no history of contraceptive pill use or herbal medication. She denied any family history of chronic or malignant diseases, and she had no history of alcohol consumption or smoking.

Clinical examination revealed asymmetrical breasts, with a large, fungating, ulcerating, and bleeding mass in the left breast that occupied the entire breast and was associated with a foul-smelling discharge. The mass had invaded the nipple-areola complex (NAC), leading to its deformation. The estimated size of the tumor was approximately 20 × 20 cm (Figure 1). In the right breast, there was a small, palpable, soft, mobile, painless mass measuring around 3 × 4 cm located behind the NAC. No palpable lymph nodes were noted in either axilla.

Our preliminary diagnosis was a phyllodes tumor versus malignant breast cancer. A 5-mm punch biopsy was performed at the clinic and sent for histopathological analysis. The patient was admitted and managed conservatively with intravenous fluids and cefazolin due to a fever spike during admission. All laboratory and biochemical parameters were within normal limits.

**Figure 2:**
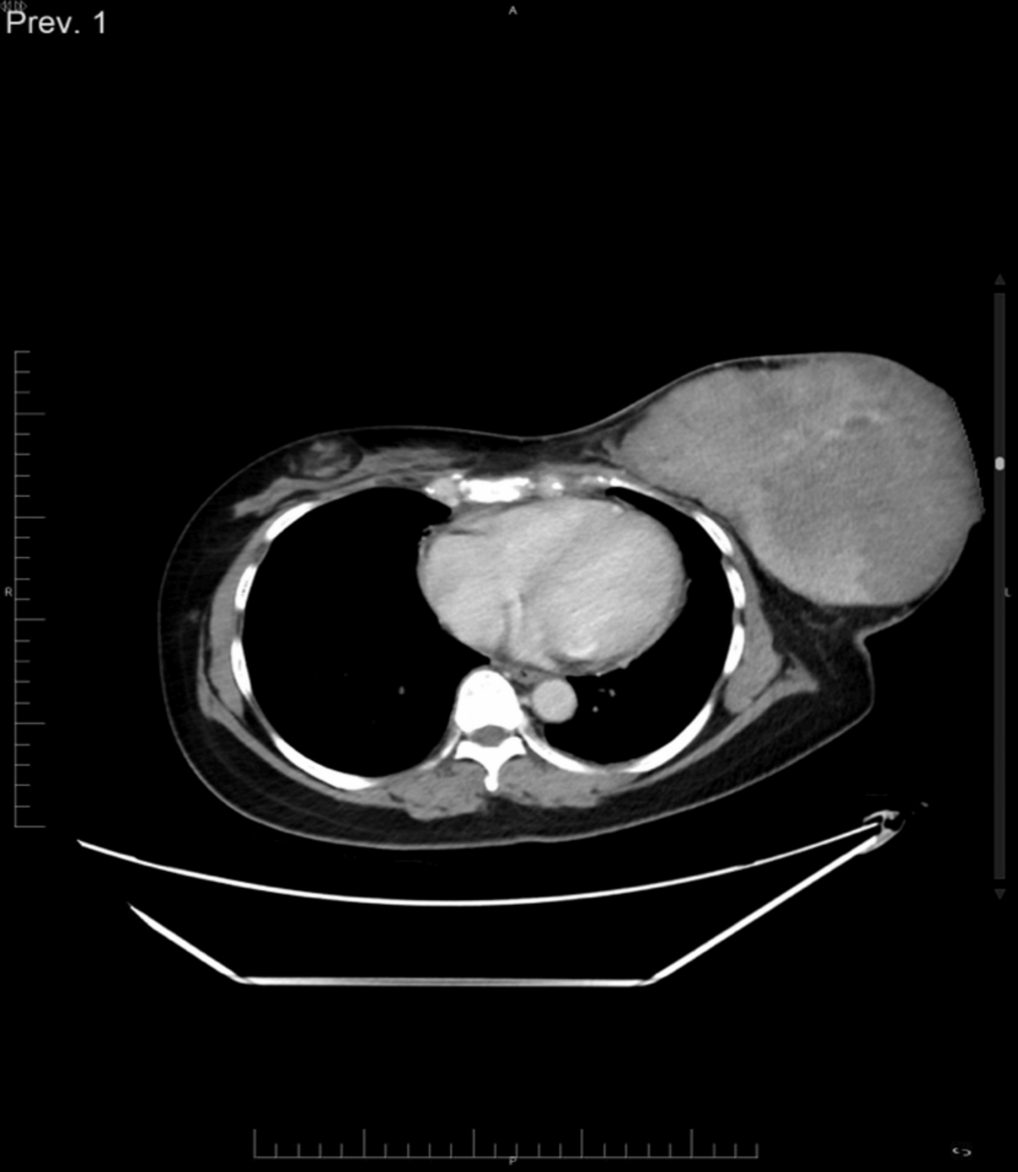
CT scan of the thorax showed large heterogeneously enhancing left breast fungating lesion involving most quadrants measuring around 17 × 12 cm

Due to ulceration and bleeding of the left breast mass, we were unable to perform a mammogram or ultrasound of that area. However, we successfully conducted an ultrasound of the right breast and bilateral axillae. The right breast showed a 3.8 × 1.2 × 3.8 cm oval-shaped lesion suggestive of a hamartoma (BIRADS 2), while the bilateral axilla ultrasound revealed no significant axillary lymphadenopathy. Given the suspicion of breast malignancy, a staging CT scan of the chest was performed. It revealed a large, heterogeneous left breast lesion involving most quadrants, measuring approximately 17 × 12 cm. The mass was in close proximity to the pectoralis major muscle, and underlying muscle invasion could not be completely excluded due to the tumor's size. The right breast also exhibited a fat-density lesion suggestive of a hamartoma measuring 3.6 × 2.1 cm, correlating with the ultrasound findings. No significant lymphadenopathy was detected on the CT scan (Figure 2).

At this point, the skin punch biopsy results were reported, showing severe mixed inflammation and granulation tissue. Immunohistochemistry for CK7 and GATA3 was negative for neoplastic cells, rendering the skin punch biopsy inconclusive. Consequently, we proceeded with a core biopsy of the left breast mass. The core biopsy revealed glandular and stromal elements, with glands lined by benign ductal epithelial cells. The stroma exhibited increased cellularity, classified as B3 atypia, which was likely benign.

An incidental finding on the staging CT scan of the pelvis revealed an enlarged, heterogeneous enhancing mass on the uterine cervix, suggestive of a leiomyoma. We counseled the patient on all findings and the proposed management plan. She agreed to undergo a simple mastectomy of the left breast and a cervical myomectomy to remove the leiomyoma, with assistance from the gynecology team.

The simple unilateral mastectomy was performed, with the wound closed by approximating healthy upper and lower flaps. Hysteroscopy and cervical myomectomy were also carried out under general anesthesia by the gynecologist, all in one setting.

**Figure 3:**
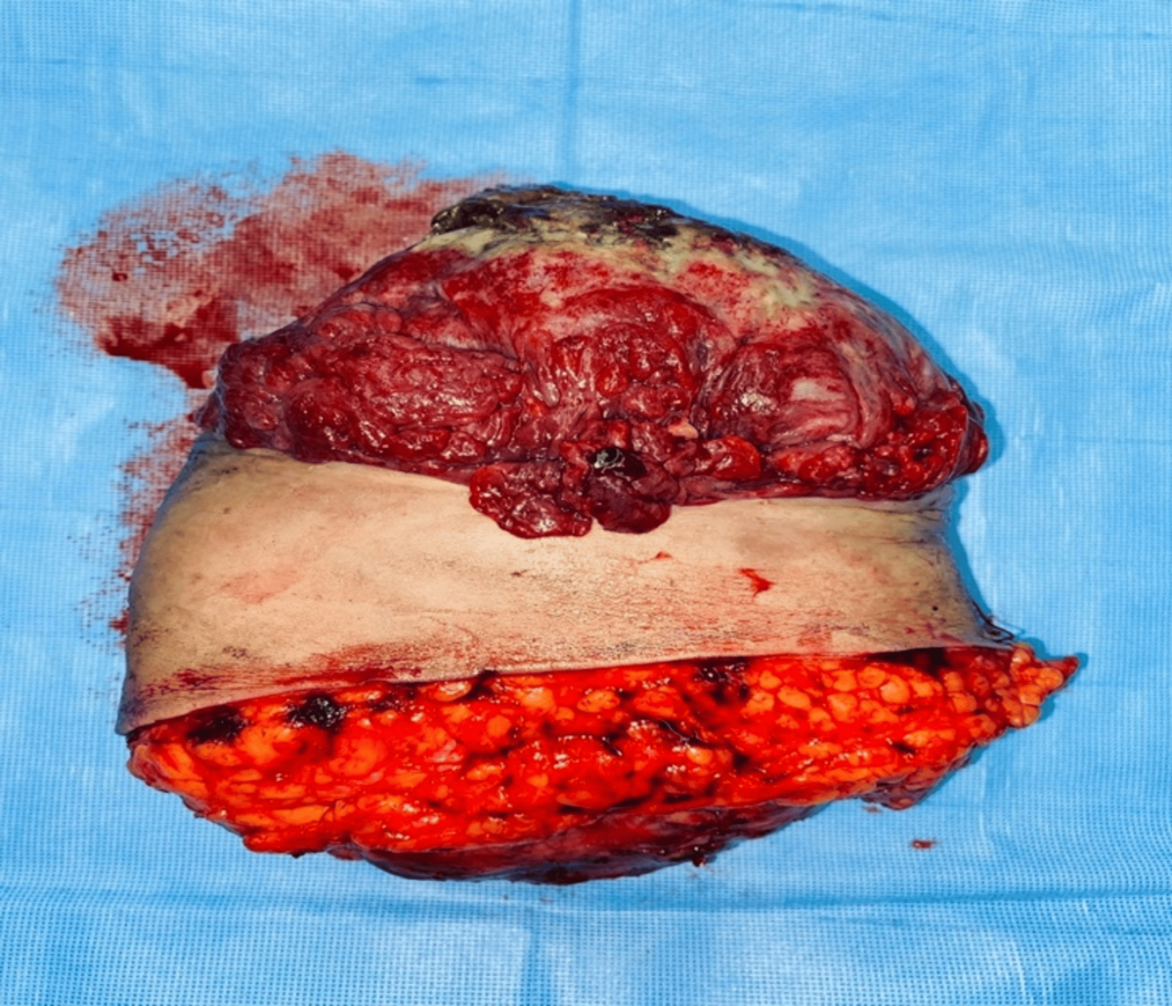
Gross specimen showed a left mastectomy with obliterated NAC NAC: nipple-areola complex.

The patient had an uneventful postoperative recovery. The final histopathology report of the breast tissue indicated a tumor measuring 17 × 15.5 × 14 cm and weighing 1,995 g (Figure 3). The report confirmed that ducts were compressed in areas and exhibited fibrocystic changes, including cystically dilated glands with apocrine metaplasia. The stroma showed mild focal myxoid changes, but no ductal carcinoma in situ (DCIS), malignancy, cytological atypia, or mitosis were observed.

**Figure 4:**
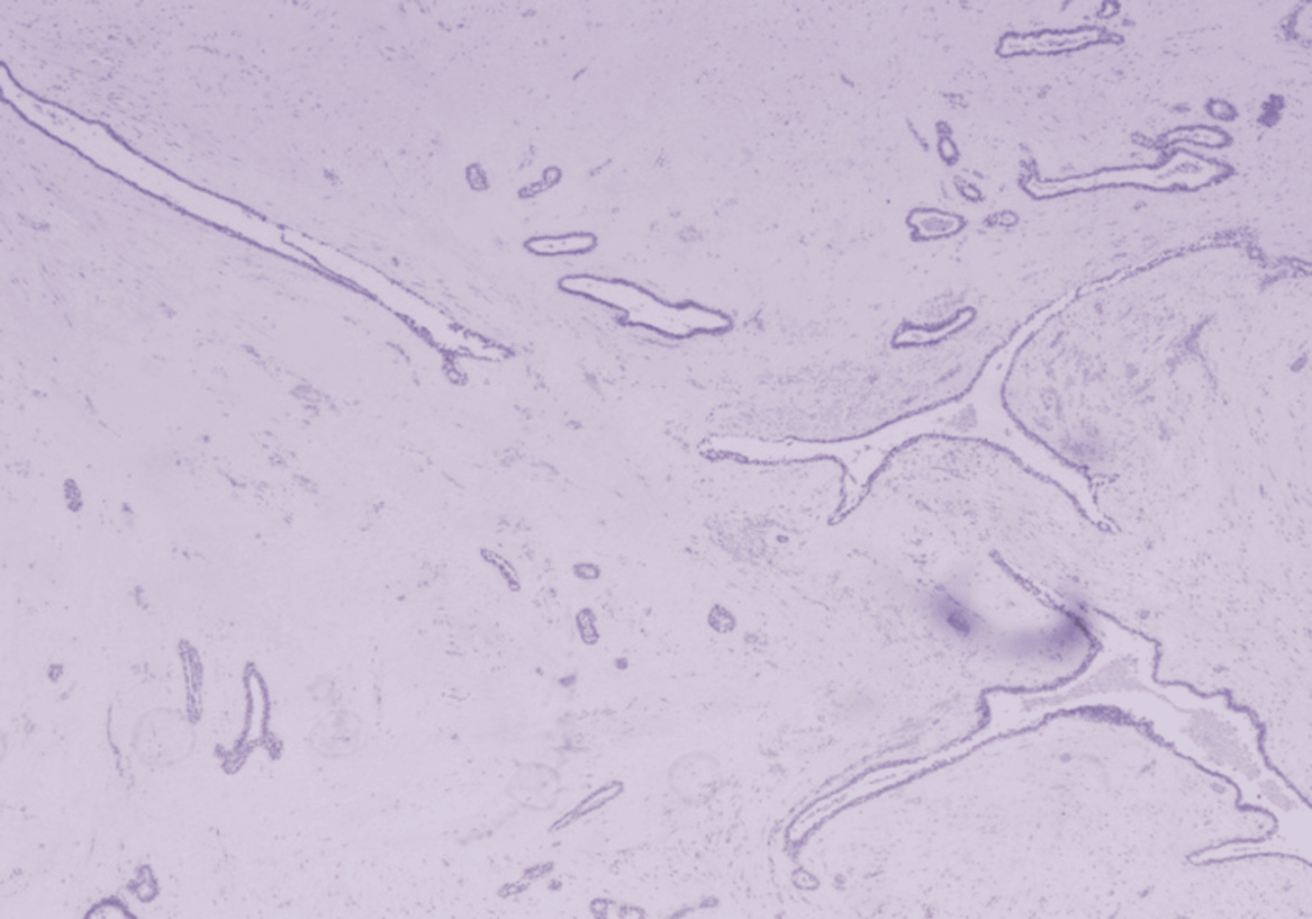
Scanner view shows the tumor with dilated and elongated ducts set in a hypocellular stroma (H&E ×40 magnification)

**Figure 5:**
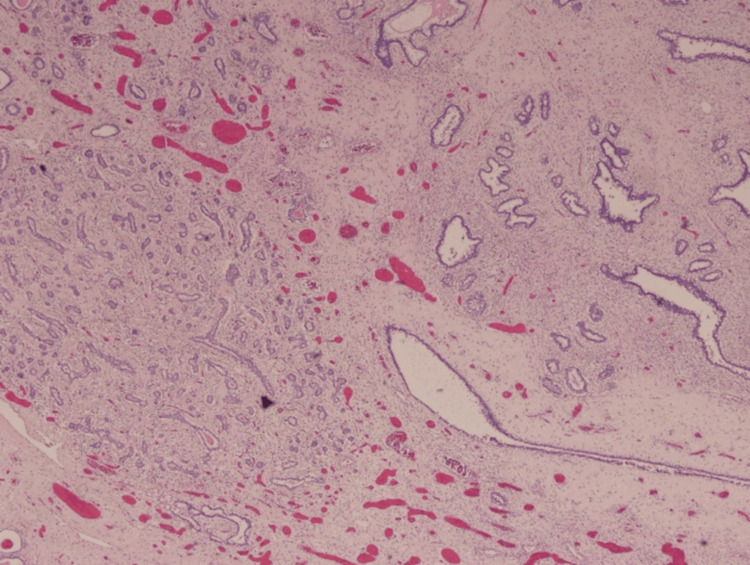
Scanner view shows tumor with areas of adenosis and an elongated duct (H&E ×40 magnification)

The final diagnosis was a giant fibroadenoma, with cystic changes and surface ulceration which was negative for any malignancy (Figures 4, 5).

**Figure 6:**
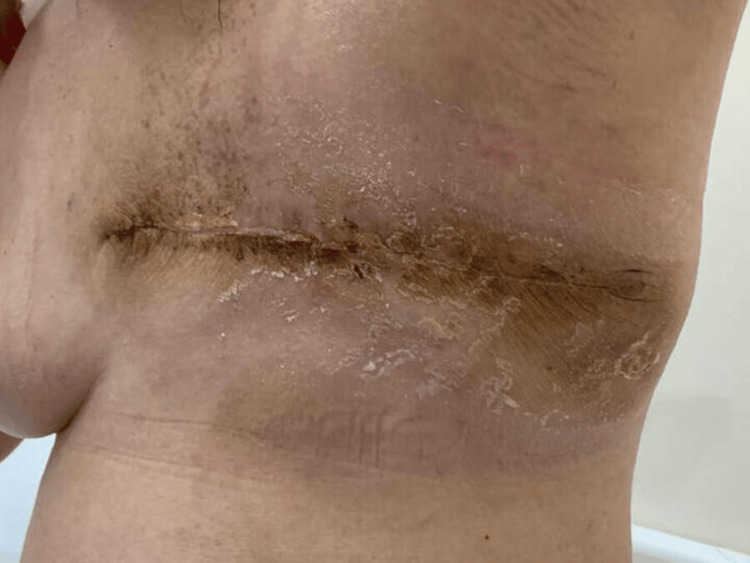
Post-left mastectomy showing healthy wound and flap healing

The uterine cervical mass measured 7 × 6.5 × 4 cm with largest dimension was diagnosed as leiomyoma. The patient was later discharged and reviewed in the outpatient clinic. She recovered and showed healthy wound healing (Figure 6).

## Discussion

Non-giant fibroadenomas of the breast are quite common, occurring in about 12% of the population [9]. In contrast, giant fibroadenomas represent only 0.5% to 2%, highlighting their rarity [10]. Today, breast awareness programs worldwide encourage women to seek early detection of breast cancer and tumors that mimic malignancy, as seen in our case. This proactive approach enables women to seek medical attention before significant symptoms develop. Unfortunately, our patient neglected the growing mass in her left breast and did not seek care earlier, allowing the tumor to progress unchecked. Fungating breast lesions are typically associated with malignancy rather than benign conditions, complicating clinical diagnosis [11,12].

Several factors in the patient's history raised concerns for a malignant differential diagnosis, including age, gender, unknown family history, and the tumor's size and characteristics [13]. While benign tumors, such as phyllodes tumors and fibroadenomas, were also considered, differentiating between these two types is challenging as they can present similarly [14,15]. However, histopathological investigation confirmed the differences between phyllodes tumors and benign fibroadenomas [15,16].

Fibroadenomas are characterized by a true capsule and hypo-cellular stromal cells, distinguishing them from phyllodes tumors [16]. Typically, fibroadenomas are managed conservatively with regular follow-ups [8]. However, in this case, the obliterated nipple-areola complex, malodor, ulceration, bleeding, and fungating features deterred the patient from seeking non-operative measures [16].

The multidisciplinary team involved in the patient's management plan unanimously agreed on surgical intervention. This team included specialists from General Surgery, Plastic Surgery, and Obstetrics and Gynecology. Leaving the lesion to grow further and worsen was considered both risky and undesirable. Additionally, the Obstetrics and Gynecology consultant concurred that performing a hysteroscopy and cervical myomectomy to excise the leiomyoma was the best approach.

The final management plan involved excising the lesion along with the involved margins [8]. We decided to proceed based on recommendations from earlier local cases at the hospital [1]. The Chinese Society of Breast Surgery (CSBrS) provided guidelines in 2021 for treating breast fibroadenomas, which stipulate that lesions should be >3 cm in size. However, these guidelines do not specifically address giant lesions (>5 cm) or those that are fungating, ulcerating, or bleeding, thus mimicking malignancy, as seen in our case [17].

## Conclusions

Giant fibroadenomas which are benign remain rare, despite the availability of numerous breast cancer awareness programs in the region. The rapid growth and worsening characteristics of the lesion prompted early consultation for the patient. We emphasized that timely intervention could lead to better cosmetic outcomes by preserving more breast parenchyma. In conclusion, we advocate for the establishment of precise guidelines at the hospital, to facilitate a smoother management process for both patients and physicians.
